# Healthcare professionals’ engagement in palliative care for patients with mental disorders: a meta-synthesis of qualitative studies

**DOI:** 10.3389/fpsyt.2026.1898822

**Published:** 2026-08-05

**Authors:** Shuting Tan, Suo Yang, Chunhua Nie, Yingying Fan, Li Zhang

**Affiliations:** The Fourth Hospital of Changsha (Integrated Traditional Chinese and Western Medicine Hospital of Changsha), Changsha, China

**Keywords:** engagement, healthcare professional, mental disorders, palliative care, qualitative

## Abstract

**Background:**

Palliative care offers solace, mitigates pain, and enhances quality of life. However, individuals with mental diseases have inadequate access to palliative care and encounter various barriers during the process. As the need for palliative care is expected to rise significantly in the future, palliative care must be considered a global health priority for healthcare professionals to upgrade care quality. This study aims to synthesize qualitative evidence on healthcare professionals’ experience of providing palliative care for patients with mental disorders, so as to identify relevant obstacles and facilitators and provide evidence reference for developing targeted intervention schemes.

**Design:**

A systematic review and meta-synthesis of qualitative studies.

**Methods:**

Qualitative studies focusing on healthcare professionals’ experience of providing palliative care for patients with mental disorders were retrieved from Chinese and English databases from database inception to April 2025. The quality of included literature was assessed by the JBI qualitative research quality evaluation standard, and convergent meta-synthesis was adopted to integrate relevant findings.

**Results:**

A total of 1258 studies were identified through database research, and 11 studies were incorporated into the review. Three analytical themes emerged from the findings:perceptions toward palliative care including; multi-level barriers in implementing palliative care and suggestions for improving palliative care services.

**Conclusion:**

Healthcare professionals show various perceptions and face complex practical obstacles when providing palliative care for individuals with mental disorders. Healthcare professionals must address cognitive biases and manage multidisciplinary competency deficiencies in routine care. Simultaneously, the promotion of tiered training, systematic service enhancement, and standardized interdisciplinary collaboration is essential to refine the service system, ensuring high-quality and dignified palliative care for patients with mental disorders.

**Systematic Review Registration:**

https://www.crd.york.ac.uk/PROSPERO/view, identifier CRD420251021339.

## 1 Introduction

Mental disorders such as depressive disorders, anxiety, bipolar disorder, schizophrenia, and substance-related disorders are long-term persistent ailments characterized by prominent comorbidities and unsatisfactory clinical treatment efficacy (1–3). These diseases impose heavy social burdens in globe. In 2019, mental disorders resulted in 418 million worldwide disability-adjusted life years (DALYs), representing 16 percent of total DALYs, which is three times the level reported in previous evaluations (4). Clinical symptoms observed in individuals diagnosed with mental illness can severely impair physical functioning and psychosocial adaptation. Despite variations in symptomatic manifestations across different diagnostic categories and coexisting conditions, prevalent manifestations including anxiety, depression, hallucinations, paranoia, and dissociation are often difficult to control and demand sustained, intensive clinical intervention (5). Bodily symptoms can stem from coexisting somatic illnesses, with coronary heart disease, diabetes, obesity and chronic respiratory diseases being the most prevalent types (6). Both clinical manifestations of mental disorders and the social prejudice toward mental health issues tend to make affected individuals feel alienated from the public. Such marginalized feelings leave them unable to integrate well into social life, which in turn further aggravates their mental distress (7). Compounding these challenges, individuals suffering from psychiatric conditions tend to have a substantially reduced life expectancy compared with the general population, with recent evidence indicating that patients with mental disorders may lose between 10 to 20 years of expected life span (8).

Palliative care serves as a comprehensive care model aimed at enhancing the life quality of patients and their families confronting life-threatening diseases. It alleviates various forms of distress via early screening, comprehensive evaluation and targeted intervention, covering physical discomfort, psychosocial troubles as well as spiritual needs (9). Given the complex care demands of mental disorders across the entire disease course and end-of-life stage, as well as the substantial burden borne by affected patients and their families, palliative care interventions are highly applicable to this patient population (10). As frontline clinical practitioners who deliver direct patient services, healthcare professionals are crucial for detecting the palliative care requirements of mental disorder patients, launching timely clinical communication, and organizing multidisciplinary supportive interventions (11). Nevertheless, individuals diagnosed with mental disorders still face severe underutilization of palliative care resources in clinical practice (12). This disparity is further compounded by healthcare professionals’ limited attention to the physical health conditions of patients with mental disorders, as well as their restricted understanding of and cooperation in palliative care delivery (13).Furthermore, patients with mental disorders frequently experience inequitable healthcare access throughout their lifespan, including the end-of-life period. Such persistent medical disparities greatly restrict their access to professional palliative care support (14). Evidence suggests that healthcare professionals’ attitudes, knowledge, and preparedness significantly influence the quality and accessibility of palliative care for this population (15). Therefore, exploring healthcare professionals’ experiences and perspectives in providing palliative care to patients with mental disorders is crucial for identifying barriers and facilitators that shape the quality and effectiveness of end-of-life support.

Examining healthcare professionals’ engagement in palliative care for patients with mental disorders is particularly suited to qualitative research, as it captures the nuanced, context-specific nature of clinical decision-making and interpersonal interactions. Although some qualitative studies have explored this topic, each is constrained by its own methodological approach, cultural context, and value framework (16), making it difficult to form a unified understanding of healthcare professionals’ psychological experiences and practical challenges. Moreover, within both domestic and international contexts, there is a lack of systematic reviews that synthesize and integrate these research findings. To address this gap, the present study adopts a meta-synthesis approach to comprehensively analyze and summarize existing qualitative evidence. Its goal is to achieve a deeper and more comprehensive understanding of healthcare professionals’ experiences and perceptions in palliative care for patients with mental disorders, thereby providing valuable reference for the development of targeted, evidence-based intervention programs.

## 2 Methods

### 2.1 Search strategy

This systematic review adheres to PRISMA guidelines strictly. A total of six English-language databases (PubMed, Web of Science, EMBASE, EBSCO, Cochrane Library, Scopus)and four Chinese-language databases (Wanfang Database, VIP Database, CNKI, SinoMed) were searched from the establishment of the database until April 2025. Search strategies were established using a combination of subject headings and free words and categorized into four areas: “healthcare professionals,” “mental disorders,” “palliative care,” and “qualitative research”. The specific search strategies for each database are provided in **Supplementary File 1**.

### 2.2 Inclusion and exclusion criteria

The inclusion criteria were formed under the guidance of the PICoS principle: (1)participant: registered healthcare professionals involved in palliative care for patients with mental disorders, such as nurses, physicians, and psychotherapists; (2)phenomena of interest: healthcare professionals’ experiences, perceptions, or insights regarding palliative care for patients with mental disorders; (3)context: hospitals, nursing homes, communities, hospice facilities, or patients’ homes; (4)study design: all types of qualitative studies, or the qualitative component of mixed-methods studies.

The exclusion criteria for studies were: (1)non-Chinese or English literature; (2)not available in full text; (3)repetitive publications or incomplete data; (4) conference abstracts, protocols, and posters.

### 2.3 Screening and data extraction

All gathered documents were imported into Endnote for the purpose of duplicate removal. Two researchers proficient in evidence-based medicine and trained in qualitative research independently performed the literature screening and data extraction, thereafter verifying each other’s work. Conflicting results would be presented to an additional researcher for collaborative deliberation to achieve a consensus final determination. The essential information gathered comprised the author’s name, publication year, country, research methodologies, research subjects, the phenomenon under examination, and the principal study results.

### 2.4 Quality appraisal

The process involved two reviewers who independently assessed the quality of the included studies using the “Quality Appraisal Standards for Qualitative Research” from the Australian Centre for Evidence-Based Healthcare (JBI) (17). Both reviewers were trained in evidence-based practice methodologies. Each criterion was assessed using a “yes,” “no,” or “unknown” response. Following the assessment, the literature was classified into Grade A, Grade B, or Grade C. Full compliance with the quality assessment criteria was assigned Grade A, partial compliance Grade B, and complete non-compliance Grade C. Grade C literature is of low quality and has a higher likelihood of bias; therefore, this study included only literature classified as Grade A or Grade B. When the two researchers’ evaluations were inconsistent, consensus was reached through discussion or consultation with a third researcher.

### 2.5 Data synthesis

During the data processing stage, the convergent integration method recommended by JBI (17) was adopted to integrate the results. This method involves extracting the main themes, potential implications, and classifications from the research conclusions, and then further summarizing and generalizing based on these contents to enhance the interpretability and comprehensiveness of the results. All sorted contents were finally consolidated to obtain highly summarized thematic outcomes.

## 3 Results

### 3.1 The basic characteristics and quality evaluation of the included literature

**Figure 1:**
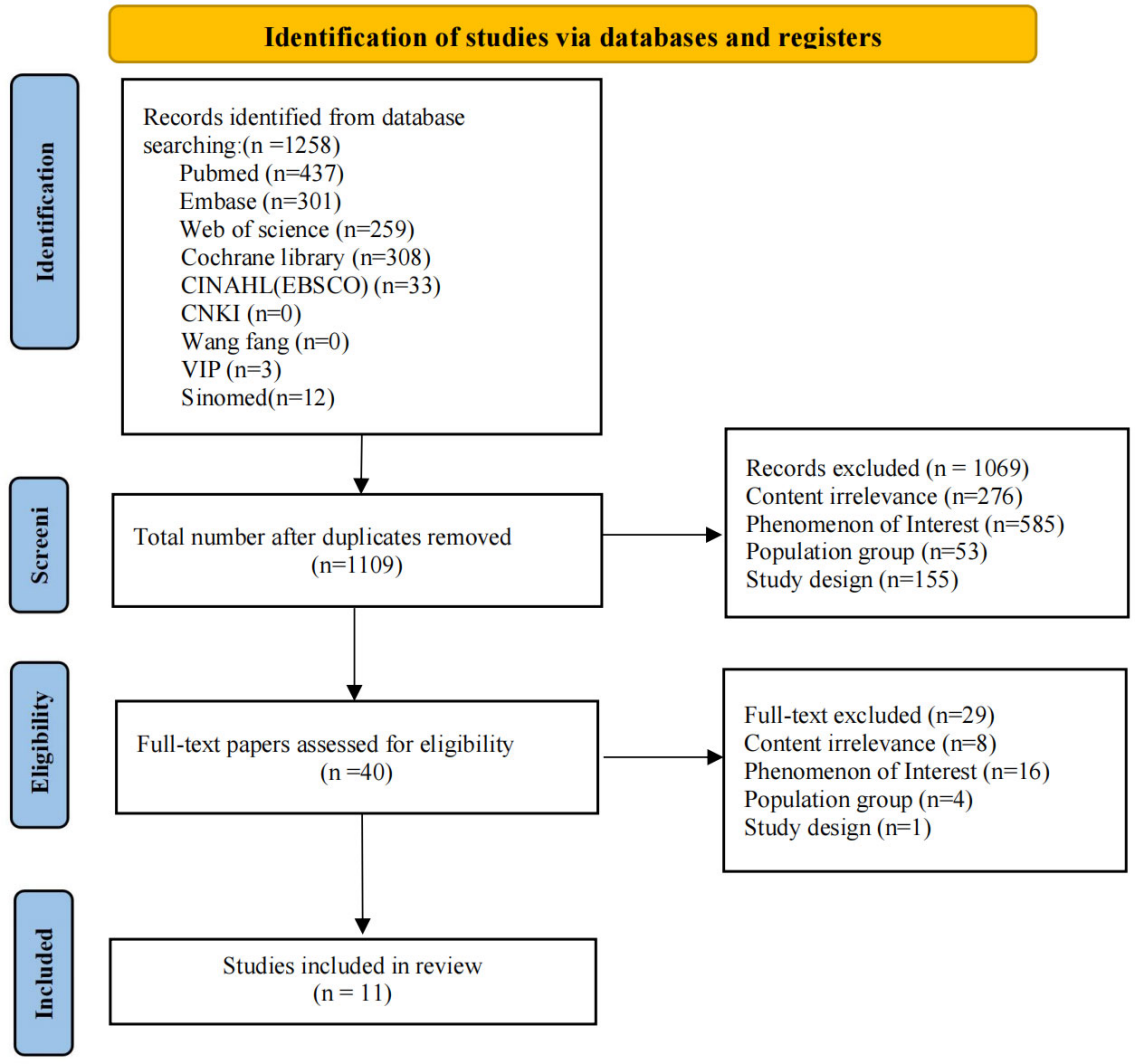
Flow diagram of the study selection process.

The initial search yielded 1258 relevant articles. Finally, 11 articles were included (7, 13, 18–27), including 11 English articles (**Figure 1**). The basic characteristics of the included articles are shown in **Table 1**. These studies were conducted in the following countries and regions: the United States (3 studies) (7, 21, 23), the Netherlands (2 studies) (19, 27), the United Kingdom (2 studies) (20, 24), Australia (2 studies) (25), Canada (1 study) (13), Iran (1 study) (18), and Denmark (1 study) (22). The data for these 11 studies were collected from 233 healthcare professionals, including nurses, palliative care nurses, mental health providers, interdisciplinary clinicians, general practitioners, palliative care physicians, clinicians and clinical nursing experts. The basic characteristics of the included studies are shown in **Table 1**, and the results of the methodological quality assessment are presented in **Table 2**.

**Table 1:** The basic characteristics of the included literature (n=11).

Authors	Year	Country	Research methods	Research subjects	Phenomenon of interest/research aim	Results
SABER et al. (18)	2022	Iran	Semi-structured interview	26 nurses	Barriers perceived by nurses when providing palliative care to patients with severe mental disorders	Two themes: poor organizational and professional infrastructure; inadequate patient and family follow-up systems
PARK et al. (13)	2022	Canada	Semi-structured interview	16 mental health care providers	Mental health care providers’ understanding and experience with palliative care	Four themes: intersectionality; limited collaboration; misconceptions about palliative care; relationships
EVENBLIJ et al. (19)	2016	Netherlands	Mixed-methods research	10 nurses	Nurses’ experiences and challenges in providing palliative care in mental health settings	Three themes: barriers in the area of physical care; barriers in the area of psychosocial care; barriers at the organizational and systemic levels
WILLIAMS et al. (20)	2003	United Kingdom	Semi-structured interview	17 clinical nursing experts	Clinical nursing experts’ perspectives on, assessment of, and management of palliative care for patients with depression	Four themes: depression is a symptom; lack of training; stigma surrounding mental illness; treatment issues
SHALEV et al. (21)	2025	United States	Semi-structured interview	45 interdisciplinary clinicians	Interdisciplinary clinicians’ perspectives on the palliative care needs of older adults with severe mental disorders	Three themes: existing palliative care models inadequately address the requirements of elderly patients with severe mental problems; physicians necessitate further training and interdisciplinary collaboration; need for the integration of mental health and palliative care services.
BONDERGAARD et al. (22)	2025	Denmark	Semi-structured interview	5 general practitioners, 6 palliative care nurses and physicians	Healthcare professionals’ experiences with palliative care for patients with severe mental disorders	Four themes: contemplations on the future; contemplations during palliative care; alterations in psychiatric symptoms in patients receiving palliative care; indeterminate positions
BAIRD et al. (23)	2021	United States	Semi-structured interview	16 healthcare professionals	Healthcare professionals’ perspectives on palliative care for patients with severe mental disorders	Two themes: Fundamental comprehension of palliative care for individuals with mental problems; contextual elements that facilitate or obstruct palliative treatment for this population.
JERWOOD et al. (24)	2018	United Kingdom	Focus Group Interviews	23 clinical staff members	Clinical staff viewpoints on obstacles to delivering palliative care for patients with mental disorders	Four themes: System architecture; patients’ problematic behaviors and convictions; professionals’ self-assurance; collaboration challenges
MORGAN et al. (7)	2016	United States	Semi-structured interview	20 nurses	Nurses’ perspectives and requirements for end-of-life care for patients with mental disorders	Six themes: the stigma from disease, the effects of mental disorders on communication and trust, dysfunctional family systems, pain and comfort care advocacy, formal support and education, and the lack of a proper location to die.
LEE et al. (25)	2022	Australia	Focus group interviews	15 clinicians	Clinicians’ viewpoints on palliative care for individuals with depression	Four themes: Overall viewpoints, obstacles and difficulties, potential remedies, healthcare systems and service tiers
ZWEERS et al. (27)	2017	Netherlands	Mixed-methods research	25 nurses	Nurses’ attitudes, actions, and requirements concerning palliative care for nervous patients	Three themes: utilization of anxiety assessment instruments by nurses, nurses’ treatments and decision-making pertaining to anxiety; nurses’ requirements in anxiety management

**Table 2:** Evaluation of methodological quality (n=11).

Included studies	①	②	③	④	⑤	⑥	⑦	⑧	⑨	⑩	Quality level
SABER et al. (18)	Y	Y	Y	Y	Y	Y	N	Y	Y	Y	B
PARK et al. (13)	Y	Y	Y	Y	Y	N	N	Y	Y	Y	B
EVENBLIJ et al. (19)	Y	Y	Y	Y	Y	N	N	Y	Y	Y	B
WILLIAMS et al. (20)	Y	Y	Y	Y	Y	N	N	Y	N/A	Y	B
SHALEV et al. (21)	Y	Y	Y	Y	Y	N	N	Y	Y	Y	B
BONDERGAARD et al. (22)	Y	Y	Y	Y	Y	N	N	Y	Y	Y	B
BAIRD et al. (23)	Y	Y	Y	Y	Y	N	N	Y	Y	Y	B
JERWOOD et al. (24)	Y	Y	Y	Y	Y	N	N	Y	Y	Y	B
MORGAN et al. (7)	Y	Y	Y	Y	Y	N	N	Y	Y	Y	B
LEE et al. (25)	Y	Y	Y	Y	Y	N	N	Y	Y	Y	B
ZWEERS et al. (27)	Y	Y	Y	Y	Y	N	N	Y	Y	Y	B

Appraisal Checklist: ① Is there congruity between the stated philosophical perspective and the research methodology? ② Is there congruity between the research methodology and the research questions or objectives? ③ Is there congruity between the research methodology and the data collection methods? ④ Is there congruity between the research methodology and the representation and the data analysis? ⑤ Is there congruity between the research methodology and the interpretation of results? ⑥ Is there a statement locating the researcher culturally or theoretically? ⑦ Is the influence of the researcher on the research, and vice- versa, addressed? ⑧ Are participants and their voices adequately represented? ⑨ Is the research ethical according to current criteria or, for recent studies, and is there evidence of ethical approval by an appropriate body? ⑩ Do the conclusions drawn from the research report flow from the analysis or interpretation of the data?

Appraisal result: Y, yes; N, no; U, unclear; N/A, not applicable.

### 3.2 Synthesis of findings

After carefully reading and analyzing 11 pieces of literature, extracted a total of 47 research results. Then grouped and combined similar results into 8 new categories, and synthesized them into 3 synthesized findings.

#### 3.2.1 Synthesized finding 1: perceptions toward palliative care

Healthcare providers generally hold a positive value orientation toward palliative care for patients with mental disorders, but this is often accompanied by cognitive biases and practical hesitation. While most recognize patients’ right to dignified end-of-life care and show empathy, they also face challenges such as limited awareness of palliative care, unclear service delivery pathways, and concerns about death. Mutual stigma between providers and patients further hinders effective care, resulting in inconsistent behavioral responses in clinical practice.

##### 3.2.1.1 Category 1: the value of palliative care and ethical commitment

Healthcare professionals widely concur that individuals with mental disorders possess the inherent right to die with dignity. This collective value and ethical obligation provide the primary impetus for their delivery of palliative care. As one interviewee put it: [“*We enter this life with dignity, and we ought to depart with dignity; it is my duty to assist patients in attaining this objective.*” *(*21)]. This shared comprehension of values subsequently manifests in proactive care practices. On one side, healthcare personnel should prioritize patients’ emotional and relational needs throughout the end-of-life phase[“*Restoring fractured relationships is integral to nursing, with an emphasis on emotional resolution and the resolution of unresolved matters at life’s conclusion.*” *(*19)]. On the other hand, they are willing to devote extra time to understanding each patient’s individual circumstances[“*This position is quite significant, so I dedicated additional time to comprehend her requirements and mental condition, while endeavoring to grasp their circumstances.*” (22)]. At the same time, healthcare providers are keenly aware of the individual differences among patients, and this awareness lays the foundation for providing personalized care. Some patients are able to accept their current situation relatively well[“*His mental health has not declined. Given the diagnosis, he is performing unexpectedly well and has come to terms with his circumstances.*” *(*22)], while others remain plagued by the disease over the long term, for example[“*Despite the efficacy of rehabilitation exercises, individuals with severe schizophrenia continue to experience enduring distress.*” (23)].

##### 3.2.1.2 Category 2: individual-level cognitive biases, attitudinal barriers, and stigma

In terms of service awareness, healthcare professionals generally do not place sufficient emphasis on palliative care and have limited knowledge in this area. Most respondents acknowledged the lack of relevant vocational training and work attention[“*Palliative treatment for eligible patients is infrequently contemplated, and talks regarding it with colleagues are seldom conducted in standard clinical practice.*” (13)]. At the same time, the choice of service location poses practical challenges and home-based palliative care is difficult to implement[“*Numerous patients reside in inadequate conditions, frequently using hostels, shared apartments, or subpar single-occupancy accommodations.*” (24)]. Meanwhile, bed capacity within healthcare facilities cannot meet the demand[“ *Some wards are appropriate for certain patients; nevertheless, there are consistently no beds available upon a patient’s arrival.*” *(*7)].

At the clinical decision-making level, complex medical conditions exacerbate hesitation among healthcare providers. Healthcare professionals struggle to identify the best intervention time for fluctuating patient conditions[“*I frequently questioned the immediacy of symptoms and opted to delay response to prevent misassessment.*” (20)]. When a patient’s condition worsens, this uncertainty is further amplified[“*As their illness intensified, the circumstances were increasingly complex, leaving me frequently uncertain about how to proceed.*” *(*21)]. Additionally, healthcare providers’ psychological aversion to death also affected their level of care to some extent[“*I appreciated working in psychiatry, particularly because there were no fatalities involved.*” *(*7)].

In terms of doctor-patient interactions, mutual stigmatization further exacerbates the gap in care. On the one hand, some healthcare providers hold fear and stereotypical perceptions toward patients with mental disorders[“*Some clinicians harbored apprehensions against psychiatric patients and deliberately refrained from engaging with them.*” (13)]. At the same time, patients labeled as “uncooperative” tend to be neglected clinically[“*Patients classified as treatment-resistant in discharge records are frequently overlooked and receive minimal follow-up care.*” (13)]. On the other hand, patients’ self-stigmatization hinders end-of-life communication[“*The majority of patients did not discuss mental health concerns and struggled to comprehend pertinent enquiries posed at end-of-life stages.*” (25)].Public preconceptions regarding mental illness, especially schizophrenia, render healthcare personnel uncertain during conversations[“*Upon the mention of an individual with a mental condition, they exhibited profound dread, reluctance to engage, and uncertainty in their response.*” *(*7)]. In addition, cultural stigma indirectly affected the accessibility of palliative care services[“*Stigmatization and social prejudice compel psychiatric patients to deny their condition and reject hospital registration, so obstructing regular follow-up and access to palliative care.*” (18)].

#### 3.2.2 Synthesized finding 2: multi-level barriers in implementing palliative care

Providing palliative care for patients with mental disorders is impeded by interconnected barriers that operate simultaneously at three distinct levels: (1) the provider level including cross-disciplinary competency gaps constrain day-to-day clinical performance; (2) the patient and family level such as communication challenges, reluctance to discuss end-of-life issues, and insufficient family support impede care engagement; the system level involving inadequate organizational resources, lack of a structured training framework, and ineffective interdisciplinary collaboration obstruct service delivery. To keep the categorization internally consistent, the three sub-categories below are organized strictly along these three levels. Together, these multi-level obstacles produce a fragmented care environment that makes it difficult to deliver consistent, person-centered end-of-life care to this vulnerable population.

##### 3.2.2.1 Category 3: cross-disciplinary competency gaps in clinical practice

When providing palliative care for patients with mental disorders, healthcare professionals commonly face the challenge of insufficient interdisciplinary expertise. In terms of symptom assessment, their ability to identify depression is relatively weak, and there is an imbalance in the allocation of relevant educational and research resources[“*Currently, there exists an abundance of teaching and research material on pain evaluation and novel analgesic drugs; nevertheless, resources pertaining to depression assessment are significantly deficient.*” *(*20)]. At the same time, there is a lack of standardized depression assessment protocols in clinical practice, and practitioners lack the necessary skills[“*We lacked the capability to screen for and manage depression, hindering systematic assessments.*” *(*25)]. There are significant knowledge barriers between specialties, and each has its own specific areas of weakness. Palliative care physicians struggle to address depression-related issues, while psychiatrists are unfamiliar with end-of-life care, making them prone to misjudgments[“*Palliative care physicians are deficient in managing depression, whereas psychiatrists typically lack experience in palliative care and frequently misread patients’ negative comments as indications of suicidal ideation.*” (25)]. Psychiatric nurses also generally lack physical care skills[“*Psychiatric nurses necessitate a robust medical basis to proficiently conduct physical examinations and oversee medical disorders.*” *(*7)].These professional shortcomings directly impact care safety and service quality, as evidenced by clinical cases[“*A patient appeared with moderate pain complaints, but we thought it was psychological. By the time a diagnosis was verified, the patient had advanced breast cancer.*” *(*19)].

##### 3.2.2.2 Category 4: patient communication difficulties and inadequate family support

Throughout the caregiving process, numerous obstacles exist at both the patient and familial levels. The inherent characteristics of mental illness can result in communication challenges for patients, hence complicating clinical assessment considerably[“*These patients are unwilling to seek care on their own, which can delay diagnosis and prolong sickness. Some patients also struggle to express their feelings, complicating the assessment process.*” *(*7)]. In addition, most patients are highly averse to topics related to death and are reluctant to engage in end-of-life discussions[“*I was reluctant to discuss death as it adversely affected my mood. I preferred to focus on my remaining life, while many intentionally evaded conversation and rejected external assistance.*” *(*22)]. For patients with a history of drug dependence, pain management also presents a significant challenge[“*Chronic drug use resulted in physical tolerance, necessitating dosages for effective pain management that typically beyond conventional guidelines.*” *(*19)].Adequate familial support is another significant concern. Numerous patients experience social isolation[“*They not only lack companionship and support from family members but also face difficulties in securing familial aid regarding treatment decisions, which further exacerbates the decline of their physical and mental health*.” *(*13)]. At the same time, the current service system generally overlooks the psychological needs of family members. Family members of patients with mental disorders also require psychological counseling and professional support, yet existing care efforts focus solely on the patients themselves, with a severe lack of complementary support services[“*The psychosocial needs of families affected by mental illness must be emphasized; yet, pertinent services are limited, complicating the provision of palliative care*.” *(*18)].

##### 3.2.2.3 Category 5: shortcomings in organizational resources, training infrastructure, and team collaboration

At the system level, three interrelated shortcomings shape the care environment: organizational resource allocation and policy support, training infrastructure, and interdisciplinary collaboration mechanisms. In terms of resource allocation and policy support, there are significant shortcomings in professional staffing, physical infrastructure, and institutional frameworks. Currently, palliative care resources are primarily directed toward cancer patients, while individuals with mental disorders have long been neglected[“*Palliative care is exclusively offered to cancer patients; individuals with mental problems are overlooked due to a deficiency of specialized professionals and dedicated service facilities*.” *(*18)].Local governments are reluctant to create pertinent palliative care units due to the industry’s characteristics[“*Most institutions are understaffed and lack the equipment to provide palliative care*.” *(*19)]. The provision of these services encounters considerable obstacles and needs political support[“*There was presently an absence of policy mechanisms endorsing palliative care for patients with mental problems, and neither national nor local governments had formulated pertinent development strategies*” (24).].

In terms of education and training, a systematic training framework has yet to be established[“*We lacked experience in palliative care, and this region did not offer master’s nursing programs, leading to a deficiency of specialized personnel.*” *(*18)]. Simultaneously, healthcare professionals typically have insufficient access to knowledge regarding palliative care[“*Our knowledge on the care services and support programs accessible in both the community and hospitals was minimal*.” *(*21)].

At the level of collaborative mechanisms, interdisciplinary cooperation faces multiple obstacles. Palliative care relies on teamwork among nurses, psychiatrists, mental health professionals, but there is a dearth of effective cooperation in clinical practice[“*The existing inadequate collaboration in psychiatric wards significantly obstructed the execution of palliative treatment*.” *(*18)]. Furthermore, discrepancies in diagnostic interpretations erode team trust[“*I doubted regarding the outcomes of psychiatric assessments; the prevalent diagnosis of adjustment disorder engenders my hesitance to aggressively pursue collaboration*.” (25)].

#### 3.2.3 Synthesized finding 3: suggestions for improving palliative care services

To improve palliative care for people with mental illness, a multi-level and multi-dimensional improvement framework is required. This includes designing tiered educational and training frameworks to address competency gaps, optimizing care delivery models and resource integration to reduce stigma and increase service accessibility, and improving cooperation through regular interdisciplinary communication. These methods aim to remove disciplinary hurdles, improve support, and enable dignified and complete end-of-life care for mental health patients.

##### 3.2.3.1 Category 6: establishing a tiered and categorized education and training system

A well-structured training system covering basic knowledge and professional skills is essential to optimize palliative care delivery. In terms of basic training, practitioners should popularize palliative care within local communities and improve relevant academic courses[“*We should utilize advertising and promotion to enhance community awareness of pertinent services, while concurrently introducing specialized courses in palliative care at both undergraduate and graduate levels.*” (23)]. From a professional perspective, cross-specialty training is required to eliminate disciplinary barriers[“*Frontline clinicians can gain psychiatric experience via clinical rotations and obtain integrated training in palliative care and psychiatry*.” (25)]. Respondents suggested that psychiatrists should deepen their understanding of palliative care and enhance the sharing of patient information across departments[“*Clinicians required good assistance to address medication apprehensions, as many hesitated to administer specific symptomatic medicines due to probable drug interactions*.” *(*21)]. Apart from physicians, psychiatric nurses should also enrich their professional reserves to cope with diverse clinical situations[“*Mastering extensive professional knowledge enabled us to address diverse clinical issues with increased composure*.” (7)].

##### 3.2.3.2 Category 7: promoting the comprehensive optimization of the care system

Optimizing the current palliative care system helps deliver better end-of-life support for patients with mental disorders, which can be realized through resource integration and model upgrading. Healthcare providers need to be familiar with available social resources and make full use of community support[“*Community resources including volunteers could provide non-pharmacological therapies independently of professional psychiatric and psychological care*.” (25)]. In terms of care delivery, medical teams are encouraged to update service strategies and focus on holistic care during the treatment[“*Clinical teams could implement a patient-centered care strategy that integrates medical therapy, mental intervention, and patient advocacy, involving social workers and family members in comprehensive care*.” (21)].

##### 3.2.3.3 Category 8: building a routine multidisciplinary collaboration mechanism

In clinical practice, professionals from different specialties need to support one another. Healthcare practitioners encountering patients with acute anxiety, who are challenging to manage and leave them feeling helpless, will actively seek assistance from mental health professionals[“*I will contact a psychologist if a patient is anxious and demands my attention, preventing me from doing my daily tasks*.” *(*27)]. Regular communication effectively fosters team cohesion and optimizes care plans[“*Weekly multidisciplinary palliative care and psychiatry conferences*.” (25)]. The integration of interdisciplinary joint rounds should be added into standard practice[“*Interdisciplinary team rounds are essential, particularly for complex patients; I consider this a critical component*.” (21)].

## 4 Discussion

### 4.1 Reducing individual-level stigma and cognitive biases to strengthen provider empathy

Although healthcare professionals generally agree that patients with mental disorders have the right to live with dignity, this study found that their palliative care practices remain hindered by multiple factors, including cognitive biases, hesitancy to provide care, and mutual stigmatization—a finding consistent with previous research (28). Research by Butler et al. indicates that healthcare providers’ negative attitudes and stereotypes toward patients with mental disorders directly affect those patients’ likelihood of receiving palliative care services (29). These findings are comprehensively comprehended when stigma is viewed not merely as an individual attitude or interpersonal contact, but also as a structural phenomenon ingrained in service organizations, professional socialization, and institutional regulations that limit access to care (30). In these circumstances, public stigma, self-stigma, and clinical ambiguity may mutually reinforce each other. Providers with inadequate experience in psychiatric palliative care may resort to diagnostic simplifications or evade challenging discussions, while patients fearing rejection may suppress their distress, refuse participation, or delay seeking assistance. This interaction elucidates why ethical consensus about dignified care does not invariably result in consistent implementation, and why the knowledge disparity between palliative care and psychiatry may exacerbate uncertainty in intricate clinical decision-making (31). Consequently, anti-stigma initiatives must extend beyond mere attitude modification. Alongside incorporating real-life instances of palliative care for individuals with mental disorders and ethical communication training into medical education, services must implement reflective supervision, regular assessment of deferred or biased clinical decisions, and accountability frameworks that render discriminatory non-referral apparent (32). Public education is crucial; yet, without structural transformation that integrates mental health populations into palliative care pathways, stigma is likely to persist in routine treatment decisions.

### 4.2 Addressing multi-level barriers through provider, patient–family, and system interventions

This meta-synthesis shows that healthcare professionals’ efforts to provide palliative care for patients with mental disorders are constrained by interrelated challenges operating at the provider, patient and family, and system levels, consistent with the findings of previous studies (33).The WHO framework on integrated, people-centered health services contends that fragmented, disease-oriented delivery models undermine continuity of treatment, referral coordination, and responsiveness to individuals with complex life-course requirements (34). This framework is especially pertinent as numerous patients in the studies presented endure serious mental illnesses or other intricate mental problems, compounded by physical comorbidities, societal disadvantages, and familial stress. At the provider level, the separation between mental health services and end-of-life care has produced independent clinical norms and training pathways for the two fields, leaving individual clinicians without the cross-disciplinary competencies required for integrated holistic care in routine practice (35); prior studies have likewise confirmed that this disciplinary segmentation is a key factor limiting the quality of palliative care for patients with mental illness. At the patient and family level, communication difficulties, aversion to end-of-life topics, and the frequent absence of family psychological support further reduce patients’ engagement with palliative services and complicate clinical assessment. At the system level, unequal medical resource allocation and insufficient policy support further limit the development of related services. Compared with the relatively complete palliative care system for cancer patients, end-of-life care for patients with mental disorders lacks unified service standards and institutional guidance, so clinical practice largely relies on personal experience, which easily leads to inconsistent service quality (36). Overall, the various practical barriers identified in this study are not isolated clinical problems, but systematic defects formed by long-term disciplinary separation and incomplete institutional construction. Consequently, targeted interventions should be organized along the same three levels. At the provider level, continuing education for on-the-job clinicians should be updated to supplement practical learning about psychiatric end-of-life care. At the patient and family level, structured psychological support and counseling services for family members should be embedded in the care pathway, and communication protocols adapted for patients with reduced expressive capacity should be developed. At the system level, relevant departments need better policy and resource planning to expand targeted palliative care resources, and unified referral standards for psychiatric patients should be formulated to smooth cross-department care handover and reduce diagnostic conflicts.

### 4.3 Establishing a three-pronged pathway of education, system optimization, and multidisciplinary collaboration

The enhancement strategies derived from participant interviews in this study are consistent with the developmental focus of global psychiatric palliative care reported in previous literature. Existing research similarly identifies educational reform, care-system optimization, and interdisciplinary collaboration as key approaches to reducing structural barriers to service delivery (37). Building on that alignment, and in parallel with the three strategy categories, the following three-pronged pathway is proposed. First, on tiered education and training, unbalanced medical investment and resource distribution across regions currently hinder the universal implementation of standardized tiered training systems, making it difficult to narrow the interdisciplinary competency gap in the short term (38).Targeted financial subsidies and resource allocation should be allocated to grassroots and undeveloped medical institutions to foster equitable development of educational training. In terms of care-system optimization, using community volunteer resources to assist clinical care currently lacks unified management rules, resulting in uncertain service quality (39). Moreover, relevant administrative departments should therefore formulate unified access and training specifications for community volunteer teams so that volunteer input can be safely integrated into the care pathway alongside social workers and family participants. Concerning routine multidisciplinary collaboration, sustained supervision and assessment mechanisms for multidisciplinary teams should be constructed to stabilize long-term collaborative operation, thereby continuously improving the overall quality and continuity of palliative care services for patients with mental disorders.

### 4.4 Improving access to essential palliative medications and prescribing capacity across specialties

The availability and regulation of essential palliative medications especially opioid analgesics represent a significant and largely underexplored barrier for patients with mental disorders. According to the Lancet Commission on Palliative Care and Pain Relief, over 80% of major health-related suffering is concentrated in low- and middle-income countries, where immediate-release oral morphine is frequently unavailable, and the lowest 50% of the world’s population receives only 1% of the morphine-equivalent opioids distributed annually (40).Clinical research indicates that fragmented regulation, stringent licensing frameworks, onerous prescribing documentation, opiophobia, and inadequate pharmacology education frequently hinder the initiation of opioid therapy (41).This problem is compounded by cross-specialty disparities in palliative pharmacology expertise. Skills such as opioid rotation, equianalgesic dosing, neuropathic pain management, and titration in the presence of complex comorbidities are more established in oncology, hospice, and specialist palliative care than in mental health practice, where formal training is often limited (42). In many settings, the prescription and dispensing of strong opioids are also restricted to clinicians with specific narcotic-prescribing licenses, while psychiatric wards and community mental health services may lack direct access to controlled-drug formularies (43). Consequently, even when a mental health team accurately identifies the need for palliative-level analgesia, access to treatment may depend on external referral pathways that are often slow, poorly coordinated, and vulnerable to failure between specialties. Patients with a history of substance use may face additional disadvantage because clinicians’ concerns about misuse, tolerance, and diversion can contribute to the undertreatment of pain (44). Taken together, these findings indicate that improving palliative care for patients with mental disorders requires not only better medication availability and regulatory support, but also stronger cross-specialty prescribing capacity and more integrated referral pathways.

## 5 Limitations of the review

This study strictly followed the JBI integration method, and the quality assessment standards set by JBI for qualitative research, aiming to conduct a qualitative evidence synthesis analysis of practical obstacles and improvement suggestions of palliative care for patients with mental disorders. However, this study undoubtedly has certain limitations. Firstly, the final included research literature was only 11 articles, and gray literature was not retrieved and included, which may lead to an incomplete range of included literature; in addition, the included research articles had different cultural backgrounds, social conditions, and policy factors, which may limit the general applicability of the research results. Moreover, most of the literature failed to clearly present the cultural and value viewpoints of the researchers, which may introduce certain biases. Finally, since the evidence synthesis is based on the synthesis of the included studies to draw conclusions, the reliability of the comprehensive results is inevitably constrained by the quality of the original studies. Therefore, in future research, more high-quality original studies should be explored to ensure the quality of the articles.

## 6 Conclusion

This study synthesized barriers and improvement approaches of palliative care for patients with mental disorders, with the objective of prompting clinical managers to attach importance to the service development for this population. Understanding existing service obstacles and feasible optimization paths is essential for standardized clinical practice. Current service restrictions stem from the combined effect of personnel ability, family condition and institutional resource allocation. To improve service quality, medical staff should fully combine practical situations of patients and families while delivering daily palliative care. Targeted improvements including tiered training, resource integration and regular interdisciplinary cooperation help break service bottlenecks, which is vital to guarantee dignified end-of-life care and improve the overall well-being of patients with mental illness.
