# Supplementary material for: Healthcare professionals’ engagement in palliative care for patients with mental disorders: a meta-synthesis of qualitative studies
Source: Front Psychiatry. 2026 Aug 5;17:1898822. doi: 10.3389/fpsyt.2026.1898822 (PMC13487522; doi:10.3389/fpsyt.2026.1898822)
Supplement: Supplementary file 1 [file SupplementaryFile1.docx]

**Supplementary material Table 1：Search strategy 1258**

**PubMed**

| **Search** | **Query** | **Items found** |
| --- | --- | --- |
| #1 | ("Medical Staff"[MeSH Terms] OR "healthcare professionals"[Title/Abstract] OR "nurse"[Title/Abstract] OR "nurs*"[Title/Abstract] OR "physicians"[Title/Abstract]) | 942,508 |
| #2 | ("Mental Disorders"[MeSH Terms] OR "mental illness"[Title/Abstract] OR "mental disease"[Title/Abstract] OR "psychotic illnesses"[Title/Abstract] OR "psychiatric disorders"[Title/Abstract] OR "schizophrenia"[Title/Abstract] OR "bipolar disorder"[Title/Abstract] OR "depression"[Title/Abstract] OR "postnatal depression"[Title/Abstract] OR "manic"[Title/Abstract] OR "mania"[Title/Abstract] OR "anxiety"[Title/Abstract] OR "social anxiety"[Title/Abstract] OR "social phobia"[Title/Abstract] OR "post traumatic stress disorder"[Title/Abstract] OR "obsessive compulsive disorder"[Title/Abstract]) | 2,003,722 |
| #3 | ("Palliative Care"[MeSH Terms] OR "hospice care"[Title/Abstract] OR "palliative program*"[Title/Abstract] OR "end of life care"[Title/Abstract] OR "palliative nursing"[Title/Abstract]) | 80,777 |
| #4 | ("Experience"[Title/Abstract] OR "Feeling"[Title/Abstract] OR "Perception"[Title/Abstract] OR "Attitude"[Title/Abstract] OR "Thought"[Title/Abstract]) | 1,515,984 |
| #5 | ("qualitative research"[MeSH Terms] OR "qualitative research"[Title/Abstract] OR "methodology"[Title/Abstract] OR "focus interview"[Title/Abstract] OR "semi structured interview"[Title/Abstract] OR "phenomenology"[Title/Abstract] OR "grounded theory"[Title/Abstract] OR "ethnographic research"[Title/Abstract] OR "mixed method"[Title/Abstract]) | 527,927 |
| #6 | #1 AND #2 AND #3 AND #4 AND #5  ("Medical Staff"[MeSH Terms] OR "healthcare professionals"[Title/Abstract] OR "nurse"[Title/Abstract] OR "nurs*"[Title/Abstract] OR "physicians"[Title/Abstract] OR "Nurses"[Title/Abstract]) AND ("Mental Disorders"[MeSH Terms] OR "mental illness"[Title/Abstract] OR "mental disease"[Title/Abstract] OR "psychotic illnesses"[Title/Abstract] OR "psychiatric disorders"[Title/Abstract] OR "schizophrenia"[Title/Abstract] OR "bipolar disorder"[Title/Abstract] OR "depression"[Title/Abstract] OR "postnatal depression"[Title/Abstract] OR "manic"[Title/Abstract] OR "mania"[Title/Abstract] OR "anxiety"[Title/Abstract] OR "social anxiety"[Title/Abstract] OR "social phobia"[Title/Abstract] OR "post traumatic stress disorder"[Title/Abstract] OR "obsessive compulsive disorder"[Title/Abstract]) AND ("Palliative Care"[MeSH Terms] OR "hospice care"[Title/Abstract] OR "palliative program*"[Title/Abstract] OR "end of life care"[Title/Abstract] OR "palliative nursing"[Title/Abstract]) AND ("Experience"[Title/Abstract] OR "Feeling"[Title/Abstract] OR "Perception"[Title/Abstract] OR "Attitude"[Title/Abstract] OR "Thought"[Title/Abstract]) AND ("qualitative research"[MeSH Terms] OR "qualitative research"[Title/Abstract] OR "methodology"[Title/Abstract] OR "focus interview"[Title/Abstract] OR "semi structured interview"[Title/Abstract] OR "phenomenology"[Title/Abstract] OR "grounded theory"[Title/Abstract] OR "ethnographic research"[Title/Abstract] OR "mixed method"[Title/Abstract]) | 81 |

**EMBASE (Ovid)**

| **Search** | **Query** | **Items found** |
| --- | --- | --- |
| #1 | 'medical staff'/exp OR 'healthcare professionals' OR 'nurse'/exp OR nurse OR nurs* OR 'physicians'/exp OR physicians OR 'nurses'/exp OR nurses | 5,452,076 |
| #2 | 'mental disorders' OR 'mental illness' OR 'mental disease' OR 'psychotic illnesses' OR 'psychiatric disorders' OR 'schizophrenia' OR 'bipolar disorder' OR depression OR 'postnatal depression' OR 'manic' OR mania OR anxiety OR 'social anxiety' OR 'social phobia' OR 'post traumatic stress disorder' OR 'obsessive compulsive disorder' | 1,767,568 |
| #3 | 'palliative care' OR 'hospice care'/exp OR 'palliative program*' OR 'end-of-life care' OR 'palliative nursing' | 131,008 |
| #4 | 'experience' OR 'feeling' OR 'perception' OR 'attitude' OR thought | 2,743,753 |
| #5 | 'qualitative research'/exp OR methodology OR 'focus interview' OR 'semi structured interview' OR phenomenology OR 'grounded theory' OR 'ethnographic research' OR 'mixed method' | 2,311,586 |
| #6 | #1 AND #2 AND #3 AND #4 AND#5 | 437 |

**CINAHL (EBSCO)**

| **Search** | **Query** | **Items found** |
| --- | --- | --- |
| #1 | SU (healthcare professionals OR medical staff OR nurse OR nurs* OR physicians OR Nurses) AND SU (mental disorders or mental illness or mental disease or psychotic illnesses or psychiatric disorders or schizophrenia or bipolar disorder or depression or postnatal depression or manic or mania or anxiety or social anxiety or social phobia or post traumatic stress disorder or obsessive compulsive disorder) AND SU (palliative care OR hospice care OR palliative program* OR end-of-life care OR palliative nursing) AND SU (Experience OR Feeling OR Perception OR Attitude OR Thought) AND SU (qualitative research OR methodology OR focus interview OR semi structured interview OR phenomenology OR grounded theory OR ethnographic research OR mixed method) | 33 |

**Web of Science Core Collection**

| **Search** | **Query** | **Items found** |
| --- | --- | --- |
| #1 | TS=(healthcare professionals OR medical staff OR nurse OR nurs* OR physicians ) | 722,061 |
| #2 | TS=(mental disorders or mental illness or mental disease or psychotic illnesses or psychiatric disorders or schizophrenia or bipolar disorder or depression or postnatal depression or manic or mania or anxiety or social anxiety or social phobia or post traumatic stress disorder or obsessive compulsive disorder) | 1,086,521 |
| #3 | TS=(palliative care OR hospice care OR palliative program* OR end-of-life care OR palliative nursing) | 74,022 |
| #4 | TS=( Experience OR Feeling OR Perception OR Attitude OR Thought ) | 3,314,603 |
| #5 | TS=( qualitative research OR methodology OR focus interview OR semi structured interview OR phenomenology OR grounded theory OR ethnographic research OR mixed method) | 1,898,733 |
| #6 | #1 AND #2 AND #3 AND #4 AND #5  https://webofscience.clarivate.cn/wos/woscc/summary/69e4d629-5706-4c40-aff0-f402c65f5514-015681f7d2/relevance/1 | 256 |

**Cochrane Library**

| **Search** | **Query** | **Items found** |
| --- | --- | --- |
| #1 | (healthcare professionals OR medical staff OR nurse OR nurs* OR physicians OR Nurses):ti,ab,kw | 119676 |
| #2 | (mental disorders or mental illness or mental disease or psychotic illnesses or psychiatric disorders or schizophrenia or bipolar disorder or depression or postnatal depression or manic or mania or anxiety or social anxiety or social phobia or post traumatic stress disorder or obsessive compulsive disorder):ti,ab,kw | 209360 |
| #3 | (palliative care OR hospice care OR palliative program* OR end-of-life care OR palliative nursing):ti,ab,kw | 9775 |
| #4 | (Experience OR Feeling OR Perception OR Attitude OR Thought):ti,ab,kw | 146543 |
| #5 | (qualitative research OR qualitative research OR methodology OR focus interview OR semi structured interview OR phenomenology OR grounded theory OR ethnographic research OR mixed method):ti,ab,kw | 64659 |
| #6 | #1 AND #2 AND #3 AND #4 AND #5  (healthcare professionals OR medical staff OR nurse OR nurs* OR physicians OR Nurses):ti,ab,kw AND (mental disorders or mental illness or mental disease or psychotic illnesses or psychiatric disorders or schizophrenia or bipolar disorder or depression or postnatal depression or manic or mania or anxiety or social anxiety or social phobia or post traumatic stress disorder or obsessive compulsive disorder):ti,ab,kw AND (palliative care OR hospice care OR palliative program* OR end-of-life care OR palliative nursing):ti,ab,kw AND (Experience OR Feeling OR Perception OR Attitude OR Thought):ti,ab,kw AND (qualitative research OR qualitative research OR methodology OR focus interview OR semi structured interview OR phenomenology OR grounded theory OR ethnographic research OR mixed method):ti,ab,kw | 308 |

CNKI

| **Search** | **Query** | **Items found** |
| --- | --- | --- |
| #1 | TKA=“医护人员 + 医疗保健专业人员 + 医生 +护士” |  |
| #2 | TKA=”精神障碍 + 精神疾病 + 精神分裂 + 双相情感障碍 + 产后抑郁 + 狂躁症 + 焦虑 +社交恐惧症 + 社交焦虑症+ 抑郁 + 强迫症 + 创伤后应激障碍” |  |
| #3 | TKA=安宁疗护 + 临终关怀 + 姑息治疗 +姑息护理 |  |
| #3 | TKA=”体验 + 感受 + 态度 + 感觉 |  |
| #4 | TKA=定性研究 + 质性研究 + 现象学研究 + 扎根理论 + 人种学研究 + 混合研究” |  |
| #4 | #1 AND #2 AND #3 | 0 |

WanFang

| **Search** | **Query** | **Items found** |
| --- | --- | --- |
| #1 | 主题:(医护人员 or 医疗保健专业人员 or 医生 or护士) and 主题:(精神障碍 or 精神疾病 or 精神分裂 or 双相情感障碍 or 产后抑郁 or 狂躁症 or 焦虑 or 社交恐惧症 or 社交焦虑症or 抑郁 or 强迫症 or 创伤后应激障碍) and 主题:(安宁疗护 or 临终关怀 or 姑息治疗 or姑息护理) and主题：（体验 or 感受 or 态度 or 感觉 or 定性研究 or 质性研究 or 现象学研究 or 扎根理论 or 人种学研究 or 混合研究） | 3 |

VIP（维普）

| **Search** | **Query** | **Items found** |
| --- | --- | --- |
| #1 | 题名或关键词:(医护人员 or 医疗保健专业人员 or 医生 or护士) and 题名或关键词:(精神障碍 or 精神疾病 or 精神分裂 or 双相情感障碍 or 产后抑郁 or 狂躁症 or 焦虑 or 社交恐惧症 or 社交焦虑症or 抑郁 or 强迫症 or 创伤后应激障碍) and 题名或关键词:(安宁疗护 or 临终关怀 or 姑息治疗 or姑息护理) and题名或关键词：（体验 or 感受 or 态度 or 感觉 or 定性研究 or 质性研究 or 现象学研究 or 扎根理论 or 人种学研究 or 混合研究） | 0 |

| **Search** | **Query** | **Items found** |
| --- | --- | --- |
| #1 | ( "医护人员"[常用字段:智能] OR "医疗保健专业人员"[常用字段:智能] OR "医生"[常用字段:智能] OR "护士"[常用字段:智能]) |  |
| #2 | ( "精神障碍"[常用字段:智能] OR "精神疾病"[常用字段:智能] OR "精神分裂"[常用字段:智能] OR "双相情感障碍"[常用字段:智能] OR "产后抑郁"[常用字段:智能] OR "狂躁症"[常用字段:智能] OR "焦虑"[常用字段:智能] OR "社交恐惧症"[常用字段:智能] OR "社交焦虑症"[常用字段:智能] OR "抑郁"[常用字段:智能] OR "强迫症"[常用字段:智能] OR "创伤后应激障碍"[常用字段:智能]) |  |
| #3 | ( "安宁疗护"[常用字段:智能] OR "临终关怀"[常用字段:智能] OR "姑息治疗"[常用字段:智能] OR "姑息护理"[常用字段:智能]) |  |
| #3 | ( "体验"[常用字段:智能] OR "感受"[常用字段:智能] OR "态度"[常用字段:智能] OR "感觉"[常用字段:智能]) |  |
| #4 | ( "定性研究"[常用字段:智能] OR "质性研究"[常用字段:智能] OR "现象学研究"[常用字段:智能] OR "扎根理论"[常用字段:智能] OR "人种学研究"[常用字段:智能] OR "混合研究"[常用字段:智能]) |  |
| #4 | #1 AND #2 AND #3 | 12 |

Sino med

Scopus

| **Search** | **Query** | **Items found** |
| --- | --- | --- |
| #1 | "healthcare professionals" OR "medical staff" OR nurse OR nurs* OR physicians |  |
| #2 | "mental disorders" or "mental illness" or "mental disease" or "psychotic illnesses" or "psychiatric disorders" or schizophrenia or "bipolar disorder" or depression or postnatal depression or manic or mania or anxiety or "social anxiety" or "social phobia" or "post traumatic stress disorder" or "obsessive compulsive disorder" |  |
| #3 | "palliative care" OR "hospice care" OR "palliative program*" OR "end-of-life care" OR "palliative nursing" |  |
| #4 | Experience OR Feeling OR Perception OR Attitude OR Thought |  |
| #5 | "qualitative research" OR "qualitative research" OR methodology OR "focus interview" OR "semi structured interview" OR phenomenology OR "grounded theory" OR "ethnographic research" OR "mixed method" |  |
| #6 | #1 AND #2 AND #3 AND #4 AND #5  (healthcare professionals OR medical staff OR nurse OR nurs* OR physicians OR Nurses):ti,ab,kw AND (mental disorders or mental illness or mental disease or psychotic illnesses or psychiatric disorders or schizophrenia or bipolar disorder or depression or postnatal depression or manic or mania or anxiety or social anxiety or social phobia or post traumatic stress disorder or obsessive compulsive disorder):ti,ab,kw AND (palliative care OR hospice care OR palliative program* OR end-of-life care OR palliative nursing):ti,ab,kw AND (Experience OR Feeling OR Perception OR Attitude OR Thought):ti,ab,kw AND (qualitative research OR qualitative research OR methodology OR focus interview OR semi structured interview OR phenomenology OR grounded theory OR ethnographic research OR mixed method):ti,ab,kw | 125 |
